# Online inside and out: upper secondary students' expectations of a mental health interactive program in Sweden

**DOI:** 10.3389/fpubh.2025.1657404

**Published:** 2025-09-23

**Authors:** Helena Holmäng, Margaretha Larsson, Rajna Knez, Kristina Carlén

**Affiliations:** ^1^School of Health Sciences, University of Skövde, Skövde, Sweden; ^2^Institute of Neuroscience and Physiology, Sahlgrenska Academy, University of Gothenburg, Gothenburg, Sweden; ^3^Department of Child and Adolescent Psychiatry, Skaraborg Hospital, Skövde, Sweden

**Keywords:** adolescent, digital media, health promotion, introductory programs, qualitative research, reflexive thematic analysis

## Abstract

**Background:**

Adolescence represents a critical developmental stage with significant implications for long-term health and wellbeing. As this period increasingly takes place within digital environments, concerns regarding adolescent mental health have intensified. Students enrolled in the Introductory Programs (IM) in upper secondary school constitute a particularly vulnerable population, exhibiting heightened risks for mental health challenges and potential future social exclusion. These factors make IM students a key population for targeted mental health promotion efforts, yet little is known about how they perceive such initiatives or what they hope to gain from participation. Accordingly, this study aimed to describe the expectations of IM students regarding the implementation of *On the Inside* in relation to their mental health.

**Methods:**

This study employed a qualitative design guided by reflexive thematic analysis. Data were collected through semi-structured individual and focus group interviews with 17 students enrolled in IM programs. The interviews were conducted at the outset of the mental health interactive program *On the Inside*.

**Results:**

Analysis yielded four themes: Establishing a balanced lifestyle as a foundation for growth; Finding focus and connection beyond the screen; Seeing yourself and others more clearly; and Building self-esteem through awareness and agency. These themes illustrate participants' perceptions of *On the Inside* as a valuable opportunity for personal reflection, emotional development, and intentional behavioral change aimed at enhancing mental wellbeing.

**Conclusion:**

The findings highlight students' aspirations for increased self-awareness and alignment between their inner experiences and external lives, including their emotional needs, personal values, and life goals. Moreover, the study underscores the relevance of fostering healthier digital habits among adolescents to support mental wellbeing and positive development. These insights may inform the development and implementation of future mental health interventions targeting vulnerable youth populations.

## 1 Introduction

Adolescence—the transitional period between childhood and adulthood—is marked by rapid physical, cognitive, and social development, all of which shape how adolescents think, feel, make decisions, and relate to their environment (1). Beyond developmental changes, it is a critical period in which health and lifestyle habits are established, influencing wellbeing across the life course (1, 2). Adolescence thus presents both opportunity and vulnerability, with mental health serving as a crucial resource for navigating change and challenges, forming a stable sense of identity, and supporting overall positive development (2).

However, recent decades have seen a troubling rise in adolescent mental health concerns globally, with increasing self-reported health complaints and perceived loneliness, particularly among girls (3). In Sweden, 24% of girls and 14% of boys report feeling lonely most of the time or always. Furthermore, 77% of girls and 46% of boys experience at least two recurring complaints weekly, commonly nervousness, irritability, low mood, headaches, and sleep difficulties (4, 5). These trends are linked to shifts in adolescents' living conditions and lifestyle habits, with digitalization playing a significant role (3, 6, 7).

Digital media, such as social media and video games, are now integral to adolescents' daily lives—one in five remain connected during the night—and it has become more common to socialize online than in person (8). These platforms can foster social connection by providing emotional support, a sense of belonging, and access to learning, entertainment, and relaxation (9). However, a comprehensive report from the Public Health Agency of Sweden (6) highlights that many adolescents struggle to limit their screen time. Among older adolescents, daily use often exceeds 7 h—primarily on smartphones—with social media being the most frequent activity, particularly among girls. For boys, video games are also prevalent, with one in five playing for three or more hours per day. Excessive screen time may displace essential activities such as physical activity, schoolwork, and sleep. These are all critical to wellbeing, as insufficient physical activity, inadequate sleep, and poor academic performance are associated with an increased risk of mental health problems (10–12). Beyond these lifestyle factors, digital media also shapes adolescents' psychological and social wellbeing. Social media, in particular, may affect self-perception and relationships through mechanisms like social comparison and negative interactions, including cyberbullying (13, 14).

Challenges to adolescents' wellbeing can negatively affect their academic performance, and the interplay between mental health difficulties and poor achievement may shape long-term educational trajectories (11, 15). Moreover, students who struggle academically face increased risks of future mental health problems, unemployment, and social exclusion—outcomes that may have broader consequences, such as reduced societal capacity and increased welfare expenditures (16–18). These risks are particularly pronounced among students who do not transition to or complete upper secondary education. In 2023, 14.8% of Swedish 9th-grade students completed compulsory school without meeting eligibility requirements for national upper secondary programs (19). These students may enroll in one of the Introductory Programs (IM), which aim to prepare them for continued education or labor market entry (20). IM students form a heterogeneous group but often experience poorer mental and physical health than their peers. They more frequently face challenges such as negative school experiences, absenteeism, learning difficulties, language barriers, and limited socioeconomic resources (21, 22).

Given this context, IM students represent a key group for health promotion efforts, with the potential to support not only their educational progression but also their overall wellbeing, both in the short and long term. One such initiative is *On the Inside*, an interactive educational program currently being implemented and evaluated in compulsory school (grades 7–9) and upper secondary schools, including IM, as well as within the municipalities' activity responsibility in southwestern Sweden. To ensure that interactive programs like *On the Inside* are relevant and effective, it is important to understand what students themselves hope to gain from participation. While student perspectives are increasingly sought in intervention research, they are rarely explored prior to implementation. By examining expectations before program delivery, this study contributes novel insights into how such initiatives are initially perceived by a key target group.

To support interpretation of the findings, this study draws on the emerging middle-range theory of transitions, as described by Meleis et al. (23). This theory offers a framework for understanding how individuals experience and navigate life transitions, and how contextual factors shape these processes. During adolescence, such transitions include both developmental (e.g., cognitive maturation, identity formation, increased autonomy) and situational changes (e.g., school transitions, new roles and relationships). These processes involve ongoing adaptation and can present both opportunities and vulnerabilities. Personal factors (e.g., attitudes, knowledge, beliefs), community contexts (e.g., schools, peer and family relationships), and societal conditions (e.g., norms, cultural attitudes, stigma) can either support or hinder healthy development. While the theory did not guide the inductive analysis, it provides a valuable lens in the discussion for interpreting the factors that may facilitate or hinder IM students' transition to adulthood. Other components of the model—such as transition properties, patterns of response, and outcome indicators—fall outside the scope of this study.

Accordingly, this study aimed to describe the expectations of students in the Introductory Programs (IM) regarding the implementation of *On the Inside* in relation to their mental health.

## 2 Methods

This study employed a qualitative design. Data were collected through interviews and analyzed using reflexive thematic analysis (RTA) as outlined by Braun and Clarke (24). The study was conducted within the framework of the project; *In the digital era, promoting adolescents' mental health through an interactive program*, which seeks to explore whether adolescents' mental health can be strengthened through participation in the educational interactive program *On the Inside*. The interactive program is designed to disseminate knowledge, reduce social exclusion related to mental health challenges, and provide adolescents with perspectives, insights, and practical tools to promote mental health.

*On the Inside* consists of five modules that address a broad range of common challenges faced by adolescents today: healthy habits; focus time; cognitive distortions; performance anxiety; and habit-forming digital design. Each module includes a dramatized film (approximately 20 min) that incorporates short segments of factual scientific information presented by experts, and leads into reflective exercises, discussion questions, and self-help technique activities. The modules are delivered in student groups, facilitated by a teacher. Guardians are also involved through informational materials and short videos accompanying each module.

### 2.1 Study settings and participants

A purposive sampling strategy was employed. Participants were students enrolled in IM across various municipalities in southwestern Sweden, all at the outset of their participation in the interactive program *On the Inside*. The exclusion criterion was a lack of proficiency in Swedish. In total, the study included 17 participants (10 boys and seven girls), aged 16–18, with one participant aged 21. All lacked grades in at least one core subject—Swedish, English, or Mathematics—from compulsory school.

### 2.2 Data collection

The interviews, three individual and five in groups (involving two to five adolescents per group), were conducted between April 2024 and January 2025. Using a semi-structured format, the interviews allowed for flexible, responsive dialogue, with follow-up questions adapted to participants' answers. Each interview began with the open-ended question, “Tell me about your expectations regarding participating in *On the Inside*,” followed by questions encouraging elaboration and nuance. The conversation gradually moved toward more specific domains such as lifestyle, relationships, and mental health. All interviews took place in person at the students' schools, either in a conference room or a study room, conducted by the author KC and recorded using a Dictaphone. Audio files were transcribed, half by a professional transcriber and half by the author HH.

Participants were given the option to take part either individually or in a group, based on their preference and comfort level. This approach was considered appropriate given the potentially sensitive nature of the topic. The combination of formats allowed for methodological triangulation, supporting a richer and more nuanced understanding of participants' expectations, as both personal reflections and group interactions informed the data (25).

### 2.3 Analysis

Data were analyzed using RTA, following the six-phase process outlined by Braun and Clarke (26), and further elaborated in their subsequent work (24). RTA aligns with this epistemological stance by conceptualizing analysis as an active and iterative process, where the researcher plays an integral role in constructing meaning rather than uncovering objectively existing themes. This approach allows for flexible and in-depth engagement with the data, treating researcher reflexivity as a resource for insight rather than a source of bias (24). RTA is suited to exploring how individuals make sense of and attribute meaning to phenomena. In the initial phase, the author HH read the data thoroughly and recorded early reflections, which informed the preliminary coding that was subsequently developed and refined through an iterative process. Coding was conducted inductively, using short descriptive phrases or meaning units, to capture meaningful segments of text without reliance on a pre-established coding framework. These codes were grouped into preliminary themes, based on both content similarity and shared underlying meanings. Themes were refined iteratively through the development of thematic maps, the reorganization of codes in the themes, and repeated comparisons with the full dataset to ensure that they accurately represented participants' perspectives and deeper patterns of meaning. Each theme was then clearly defined and named to reflect its central meaning. This process resulted in four themes. The findings emerged through close engagement with the empirical data and critical reflexivity regarding the author's positionality, prior knowledge, and potential interpretive influences.

While data saturation was not used as a guiding principle for this study, the dataset was considered sufficient for the analytical purpose and for developing rich, well-supported themes. This decision aligns with the principles of reflexive thematic analysis, which emphasize depth, nuance, and meaning-making over data redundancy (24).

To support critical reflexivity and enhance the credibility of the analysis, the developing themes were iteratively discussed within the research team. These collaborative dialogues served as a form of analytic validation, deepening interpretive engagement and contributing to a more nuanced and well-supported reading of the data (24).

HH holds a master's degree in public health and has professional experience in research on neurodevelopmental disorders in children and adolescents. KC is a District Nurse and School Nurse with a PhD in Health and Care Sciences. ML is also a District Nurse and holds a PhD in Nursing Sciences. RK is a senior physician and psychiatrist engaged in psychiatric research, with clinical experience as a licensed psychotherapist. RK also holds a PhD in Biomedicine, in the field of Psychiatry.

### 2.4 Ethical considerations

Ethical approval for the project *In the digital era, promoting adolescents' mental health through an interactive program* was granted by the Swedish Ethical Review Authority (diary IDs: 2024-03058-02 and 2023-06988-01). The current study adhered to ethical guidelines for research in accordance with the Swedish Research Council's (27) principles of good research practice, with particular emphasis on the principle of individual protection, ensuring participants' rights, privacy, and safety throughout the research process.

Informed consent was obtained after participants received both written and oral information explaining the study's purpose, the management of personal data, and their right to withdraw at any time.

## 3 Results

The reflexive thematic analysis generated four themes (Table 1) that reflect students' expectations of the interactive program *On the Inside* in relation to their mental health.

**Table 1:** Overview of themes from the analysis with summary descriptions and illustrative quotations.

**Theme**	**Summary**	**Illustrative quotation**
Establishing a balanced lifestyle as a foundation for growth	Students anticipated that by gaining self-awareness and establishing healthier routines through the interactive program, they would be better equipped to manage daily life and work toward their aspirations	“*I think it could help create better routines, like spending more time outside, sleeping better, and cutting down on screen time. That could make you feel better, and like, become a better version of yourself.”* (Interview 7)
Finding focus and connection beyond the screen	Students expected the interactive program to help them manage digital habits more mindfully and strengthen their sense of presence and connection with both themselves and others	“*We let go of reality – we don't really live in it. We don't focus on the present, we just sit with our phones and scroll. It's crazy… This could help us get better, to become a little more social.”* (Focus group 3)
Seeing yourself and others more clearly	Students believed the interactive program could support deeper self-awareness and understanding of others, encouraging both personal wellbeing and a more open, socially aware way of engaging with others	“*It might be helpful because it gives you time to stop and think – about how you react to things and what you could do differently. About what's going on with the people around you…”* (Interview 8)
Building self-esteem through awareness and agency	While students expressed mixed expectations, many saw the intervention as a first step toward self-understanding, self-acceptance, and confidence	“*1: You might start seeing things more positively… try to make better choices – like planning ahead, getting a job, and managing life*. *2: Yeah… and not be afraid to show who you really are.”* (Focus group 2)

The table presents the four themes generated through reflexive thematic analysis, accompanied by concise summaries and illustrative quotations.

### 3.1 Establishing a balanced lifestyle as a foundation for growth

The students expected that *On the Inside* could support them in developing greater self-awareness of their needs and habits, helping them create the conditions for a more sustainable and manageable daily life. They anticipated that engaging with the interactive program would help them improve routines, manage their time better, and make healthier choices—steps viewed as important not only for improving wellbeing and coping with everyday challenges, but also for becoming more confident, responsible, and better equipped to move toward their aspirations.

“*I've heard adults say that it's important to eat, sleep, exercise, and so on. It's true, but I hadn't really taken it seriously before. After we watched the first film, I understood why and started thinking about it more. Now I want to get a better handle on my routines.” (Interview 6)*

This reflection illustrates how *On the Inside* was expected to deepen students' understanding of the reasons behind established health advice, rather than simply restating its importance. Building on this new awareness, students reflected on their current routines and their consequences—hoping for change through the interactive program.

“*I think it can affect how we spend our time. Like getting enough sleep. Without it, everything just gets harder. Your brain doesn't work, you can't get the grades you need, school and family life suffer—you don't have the energy to do anything. By working with the interactive program, you can see what you're doing, what you need, and what you have to change to feel better.” (Focus group 1)*

Students emphasized that unmet basic needs like sleep affect both physical health and broader aspects of life, such as academic performance, relationships, and overall wellbeing. They expected the interactive program to help them recognize and reflect on their habits, providing tools for making conscious changes. Not only a way of improving daily functioning, but also as a step toward personal growth and future goals, supported by greater self-awareness and a stronger sense of agency.

The students linked physical needs like eating not only to mood regulation, but also to decision-making and risky behavior. One student reflected on how irregular eating contributed to irritability and poor decision-making, such as “*driving illegally with the EPA,”* noting that “*when you haven't eaten, you don't really think about what could happen,”* and “*afterward, you're not exactly proud of yourself.” (Focus group 4)*. (EPA is a type of vehicle adapted for young drivers in Sweden). Importantly, the student's reflection—feeling “*not exactly proud”*– suggests emerging self-awareness tied to self-image, revealing a conflict between current behavior and personal values. Many students expressed clear reasons and motivations to develop healthier routines and hoped that the interactive program would support this process—providing a more stable foundation for making choices aligned with their goals and fostering a more positive self-concept. The idea that small, intentional changes could lay the groundwork for both immediate wellbeing and becoming the person one aspires to be. In this context, *On the Inside* was seen as both a starting point and a helpful guide for reflecting and shaping healthier, more purposeful ways of living.

### 3.2 Finding focus and connection beyond the screen

The students expected that the interactive program could help them respond to the effects of constant digital engagement, particularly through social media and smartphones. They described how their media use disrupted their ability to rest, focus, and connect—with both themselves and others. Some likened their habits to addiction, describing a loss of agency and a sense of “*dissolving”* into the lives and opinions of others online. While most voiced hope that the interactive program would support them in developing more mindful digital habits, others were less certain whether change was possible. Students also hoped the interactive program would restore a sense of presence, encouraging more face-to-face interaction with peers and improved communication with parents. Thus, the interactive program was anticipated as a tool for regaining clarity and fostering more grounded relationships. Students frequently described how digital distractions made it difficult to concentrate, even on things they considered important, which highlight awareness of how digital distractions affected their focus. Therefore, they expected the interactive program to prompt further reflection and motivate behavior change and encourage to confront and rethink ingrained digital habits. Students also noted how constant digital stimulation depleted their mental resources, hoping the interactive program would facilitate change.

“*I think it's going to help me focus more, and to give me better patience because my brain won't be burnt out by, like, the constant dopamine you get from all the TikToks you see. Having the awareness that you should, like, treat your mental health with kindness is really helpful, it's going to lead to a lot of good.” (Interview 7)*

Similarly, this student was aware of the mental strain caused by digital overstimulation from short-form content like TikTok, connecting this issue to a broader need for self-compassion. Their emphasis on “*treating your mental health with kindness”* indicates managing digital habits involved not just improved focus, but also building a kinder, more sustainable self-relationship. In this way, the interactive program was expected to support not just behavior change, but also a shift in how students relate to their mental health.

While others voiced clear hopes for positive change, some students expressed more uncertainty—even as they described how digital engagement deeply affected their sense of self.

“*Maybe it can have an impact. I use social media, but I don't post anything myself. I still scroll—even though I don't really like it. It's like an addiction. Even when it's about positive things, you still get affected negatively. It's like you create memories from other people's lives, get influenced by their opinions. Especially when you're young and don't really know who you are yet. You get affected by everything, all the impressions. It's like you're dissolving. I keep scrolling even when I don't want to… but it's hard to actually do something about it.” (Interview 6)*

This quote captures the emotional complexity students associated with social media use. While the student acknowledged its negative effects, they also described feeling unable to disengage—likening it to an addiction. Social media was seen as blurring identity boundaries, especially during a formative life stage. Their reflections on “*creating memories from other people's lives”* and “*dissolving”* into external impressions highlight how social media can blur the boundaries of identity, particularly during a formative stage of life. The tentative hope that “*maybe it can have an impact”* conveys a desire for change, but also uncertainty about whether real change is possible. This reflection illustrates how students hoped the interactive program might support them in breaking unhelpful digital habits, possibly helping them to feel more grounded in themselves.

Others similarly reflected that constant digital use disrupted their ability to remain present, with scrolling hindering real-life connection.

“*1: We let go of reality—we don't really live in it. We don't focus on the present; we just sit with our phones and scroll. It's crazy… This could help us get better, to become a little more social. We're getting pretty antisocial these days, so I hope it can help with that*.

*2: Yeah, to talk and actually be with each other.” (Focus group 3)*

This quote illustrates how students experienced a sense of disconnection from the present due to habitual phone use, describing the behavior as “crazy” or out of control. They framed phone use not only as a personal distraction but also as a barrier to meaningful, in-person interaction. The hope that the program might help them “talk and actually be with each other” reflects a broader desire not merely to reduce screen time, but to foster everyday social presence and connection.

### 3.3 Seeing yourself and others more clearly

The students expected the interactive program would help them better understand themselves, reflect on their emotions and behaviors, and increase awareness of others' thoughts and feelings. Some anticipated that this insight could support their mental health by clarifying unmet needs or emotional patterns. Others hoped the interactive program would help them shift perspective, enabling them to interpret situations and relationships more thoughtfully. Students also expected it might foster greater openness—making it easier to talk about personal challenges and encouraging more supportive interactions among classmates.

The students expressed a desire of deeper self-understanding. They described a gap between their efforts and results, suggesting a sense of understanding what's actually needed for them to improve mental health. The hope that the interactive program could reveal “*what I'm missing”* highlights the expectation that it might offer not just tools, but insight into unmet emotional or psychological needs. Some students reflected on how a better understanding of their own mental health could make it easier to talk to others about personal struggles. They viewed self-understanding as a pathway to greater social openness. Importantly, they also described how this openness might spread—as others developed a better understanding of their experiences, they too might feel more comfortable opening up about personal concerns.

In addition to encouraging openness, students expected the interactive program to support broader self- and social reflection.

“*It might be helpful because it gives you time to stop and think*—*about how you react to things and what you could do differently. About what's going on with the people around you, how they're doing, and so on. What you can do, what you shouldn't do, and what you can and can't influence.” (Interview 8)*

This quote illustrates how students viewed the interactive program as an opportunity to pause and reflect on emotional responses and how they perceive and relate to others. The student's reflection of what one “*can do,” “shouldn't do,” and “can't influence”* suggests a growing awareness of personal responsibility, boundaries, and the complexities of social interactions. In this way, the interactive program was expected to foster more thoughtful and balanced approaches to navigating everyday situations—particularly within school environments—both internally and interpersonally.

“*We've kind of touched on it before, but never really gone into it. The things on the inside*—*the things you can't see… The interactive program can make things more visible. Then maybe… like, people might dare to open up if they recognize themselves in the films. And even those who don't recognize themselves might become more aware of others… and of how they themselves might affect others without even realizing it.” (Interview 6)*

This reflection suggests that the student saw the interactive program as a way to bring attention to aspects of mental health that are often unspoken or hidden—“*the things on the inside.”* By making these experiences more visible, particularly through the films, the interactive program was expected to create safer conditions for recognition, empathy, and openness regarding mental health within school environments. Students expressed hope that visibility might reduce feelings of loneliness and make it easier for individuals to share their struggles. At the same time, students who didn't personally relate to the content could still become more aware of peers' experiences and consider how their own behavior might affect others.

The students acknowledged that engagement with the interactive program might vary and that not everyone would immediately see its relevance. However, working with *On the Inside* could ultimately lead to greater social consideration—suggesting that conversations and differing perspectives might be part of the process.

### 3.4 Building self-esteem through awareness and agency

This theme captures students' expectations regarding self-esteem—what shapes it, how it might shift, and whether *On the Inside* could support change. Some students viewed self-esteem as rooted in early experiences or internal thought patterns, expressing doubt that an outside interactive program could influence it. Others anticipated that the interactive program might help by encouraging greater self-awareness, forming healthy habits, or letting go of external pressures, such as social media. Across students' reflections, self-esteem was linked not only to personal mindset but also to daily actions, social validation, and identity. For many, *On the Inside* was seen as a starting point for making intentional choices and by small steps moving toward greater self-direction and confidence.

Some students believed self-esteem was shaped early in life, making it difficult to alter. However, with sufficient motivation, there was a cautious openness to the possibility of change through the interactive program: “*but if you really want it, then maybe” (Focus group 5)*. Others emphasized that meaningful change must come from within, framing self-esteem as something shaped primarily through personal mindset and internal reflection. The students acknowledged that while the interactive program might offer useful prompts for reflection, it ultimately cannot change how someone feels. This view reflects a strong sense of personal agency, suggesting the value of the interactive program lies not in its direct influence, but rather in its potential to support internal processes already underway.

Other students viewed *On the Inside* as a starting point, recognizing that change can be difficult, especially for individuals already struggling with low self-esteem. This captures a realistic yet hopeful perspective, viewing changes in persistent thought patterns as requiring sustained effort. Thus, the interactive program was seen not as a quick fix but a meaningful first step—if supported by personal commitment. This perspective was echoed by other students, including one who anticipated that the interactive program could, as a first step, “*help us figure out who we are” (Focus group 3)*, suggesting a link between self-esteem and identity formation. *On the Inside* was thus viewed as a potential guide for developing a stronger sense of self.

“*It will definitely help. Because if you stop focusing on likes or followers on Instagram, you drop the insecurity of thinking “why am I not getting more likes?”. You kind of let go of a whole bunch of negatives. And it could be about other things too*—*like hanging out with friends or just having time to think for yourself. It's basically like meditating. You get a fresher way of thinking and don't get trapped in a dark sphere of your mind. I think the methods we learn are going to help people's self-esteem.” (Interview 7)*

The student emphasized how stepping away from external validation could help reduce feelings of insecurity and promote more self-directed thinking. They also identified other supportive aspects, such as spending time with friends and having space to reflect. By comparing this shift to meditation, the student anticipated that the interactive program might foster mindful self-awareness, thereby disrupting harmful thought patterns that contribute to low self-esteem.

Several students expressed hope that the interactive program might encourage a more positive mindset about themselves and their future. One participant reflected that it might help them “*start seeing things more positively… try to make better choices*—*like planning ahead, getting a job, and managing life,”* while another added the importance of “*not being afraid to show who you really are” (Focus group 2)*. These reflections linked positive thinking and intentional action with a sense of confidence, self-worth, and agency, highlighting how the interactive program was seen as a support for both personal growth and self-acceptance.

“*I think it can have a positive impact on self-esteem. Because when you do something good, you want to keep doing it*—*it kind of becomes a habit. And then I feel like I've done something good today, like I can go to sleep calmly and not feel like a loser.” (Interview 8)*

The students expected their participation in On the Inside to support self-esteem by encouraging everyday choices that build confidence and a sense of control. They described how doing something “*good”*—in this case, beginning to form healthy habits like working out—created momentum and offered a sense of personal validation. Rather than viewing self-esteem solely as internal, students understood it as something that could be shaped through intentional, everyday actions.

## 4 Discussion

This study explored IM students' expectations regarding the implementation of the interactive program *On the Inside*, in relation to their mental health. Reflexive thematic analysis resulted in four themes, capturing how upper secondary school students in Sweden anticipated the interactive program could support both individual and relational dimensions of mental health—ranging from healthier routines and more mindful digital habits to a mindset that promotes self-esteem, and greater self- and social awareness.

Students' expectation that *On the Inside* would help them develop healthier routines aligns with existing research emphasizing the crucial role of basic lifestyle factors—such as sleep, nutrition, and physical activity—in adolescent mental health (10, 12, 28). They also reflected on their current habits and the impact of unmet needs, including irregular eating, inadequate sleep, and a lack of restorative activities like time outdoors—findings that mirror the experiences of IM students in Warne et al.'s (29) study. Moreover, similar to the participants in Warne et al.'s research, they recognized these factors as essential to both mental and physical health, linking them to their day-to-day functioning and academic success. This reinforces the well-established connection in the literature between balanced lifestyle habits, mental wellbeing, and school performance (11, 30, 31).

While students were already somewhat aware of these foundational behaviors, they expected the interactive program to deepen their motivation by explaining the reasoning behind common health advice—making it more personally meaningful in light of their wellbeing, goals, and aspirations. This aligns with research showing that knowledge alone rarely drives behavior change. However, when paired with motivational strategies that connect information to students' values, experiences, and readiness to change, information can enhance intrinsic motivation and foster a sense of ownership over the process (28, 32).

Viewed through the framework of Transition Theory (23), these reflections suggest that a balanced lifestyle plays a key role in supporting healthy transitions. The students' narratives also emphasize self-awareness, deeper understanding (beyond surface-level knowledge), and the ability to align health behaviors with personal values and long-term goals as key supports for self-directed change. Thus, initiatives like *On the Inside* may foster healthier development by enabling the internal conditions needed to navigate transitions with greater agency and resilience.

In addition to expressing expectations for a balanced lifestyle that meets basic needs, the students voiced a clear need for support in managing their digital habits—demonstrating a critical awareness of how digital media affects their everyday life. Their accounts align with existing research showing that smartphone and social media use can negatively impact adolescents' attention, wellbeing, and self-perception (14, 33, 34). Many also felt that time spent online often came at the expense of real-world relationships. Meanwhile, prior studies emphasize social media's potential to foster both new and existing connections among adolescents (9, 35). Steinsbekk et al. (36), for instance, found that teens who spend more time on social media also tend to spend more time with friends offline. However, other research shows that simply having smartphones present during face-to-face interactions can diminish engagement, attention, and enjoyment—potentially weakening relationships over time (13, 37). Rather than a contradiction, this suggests that compulsive smartphone use—such as mindless scrolling even when with others—can undermine interpersonal connection. This was echoed in the current study, where students described how they would “just sit with our phones and scroll” in each other's company, drawing comparisons to addiction. Taken together, these findings underscore the importance of students' hope that the interactive program would help them achieve balance—where digital media supports relationships without compromising real-world presence and connection.

From the perspective of Meleis et al.'s Transition Theory (23), students' reflections on digital media use highlight several constraining conditions for healthy development. On a personal level, the compulsive nature of digital engagement appeared to fragment attention, reduce presence, and undermine students' ability to develop a stable sense of identity and emotional resilience. Their reflections also suggest that digital engagement acts as a constraining community condition, as it limits real-life social presence with both peers and parents—potentially hindering the formation of supportive relationships and the development of essential social skills. Furthermore, digital behaviors are deeply embedded in adolescents' social and cultural contexts, reflected in participants' collective framing (“we”) and their recognition of implicit expectations around online performativity. In this sense, digital engagement may also constitute a constraining societal-level condition during adolescence. Unlike previous studies that identified potential benefits of digital engagement, such as supporting social connection (9, 35), the narratives in this study did not reveal clear facilitating conditions in students' current digital environments. Still, their hope that the interactive program could help them develop more mindful digital habits points to the importance of creating spaces for reflection, self-awareness, and intentional disengagement from digital stimuli—supporting adolescents in becoming more self-connected and truly “online” in their real-world lives.

Beyond their hopes of becoming more present in real life, students also anticipated that gaining greater knowledge and understanding of mental health could strengthen peer relationships and classroom cohesion through increased awareness, openness, and consideration. Research on school-based mental health interactive programs supports the potential for these expectations to be realized, showing that improving students' mental health literacy can foster empathy, reduce stigma, and promote open dialogue by creating a shared language and opportunities for vulnerability (38–40). Moreover, participants in this study emphasized the importance of recognition and relatability in the films—an observation that aligns with research showing that mental health content is perceived as more meaningful and more comfortable to engage with when it is relevant and relatable to students (38, 41). The *On the Inside* films, which follow relatable characters in familiar contexts, may therefore enhance engagement and comprehension, enabling deeper understanding. In line with students' expectations, studies suggest that such understanding can lower barriers to discussing mental health issues and increase both awareness of behaviors that support wellbeing and the motivation to adopt them (39, 40). Importantly, the belief that *On the Inside* could support students' mental health and foster a more supportive classroom climate underscores its relevance in the specific context of IM –particularly given the link between these factors and academic outcomes—which may ultimately support students to complete their education and progress in life (11, 15, 42, 43).

Applying Transition Theory (23) to this theme, students' reflections suggest that limited emotional insight and the internalized nature of mental health difficulties can act as constraining conditions in their developmental transitions. Difficulties in recognizing one's own needs, combined with uncertainty around how to share inner experiences, may hinder both emotional development and the ability to form meaningful social connections. Moreover, some participants' narratives suggest that mental health remains a somewhat delicate subject among peers, reflecting unspoken social norms that may discourage vulnerability and limit the emotional safety needed for supportive interactions. At the same time, students' expectations indicate that the interactive program could introduce facilitating conditions—both at the personal and interpersonal level—by supporting self-awareness, emotional reflection, and perspective-taking. This highlights the importance of fostering environments where emotional experiences can be recognized, articulated, and met with understanding—aligning with existing research that identifies supportive settings, such as schools, as key to adolescents' healthy transitions (42–44).

In the final theme, students reflected on self-esteem and how it might be influenced by participating in *On the Inside*. Their expectations varied: while many held a realistic yet hopeful outlook, others expressed doubt about whether meaningful change was possible—viewing self-esteem as largely shaped early in life or too complex to shift without sustained effort. This belief aligns with findings from Berg et al. (45), where adolescents initially viewed self-esteem as complex and fixed, but later began to see it as more understandable and open to change. It is possible that participants in the current study may experience a similar shift as they gain insight into the factors shaping self-esteem, potentially fostering greater hope and agency. Nonetheless, across students' reflections, they already acknowledged various influences on their self-esteem, such as the need for external validation through social media and the importance of aligning their actions with personal values and goals. These reflections align with research highlighting the multidimensional nature of self-esteem, shaped by personal, social, and cultural factors (46–48). Within this broader framework, school environment and academic performance are also known to influence adolescents' self-esteem, particularly relevant in the IM context (49, 50). Failing to meet eligibility for upper secondary school may be perceived as a personal failure and can negatively affect self-esteem. Poor academic outcomes can also diminish hope for the future—a pattern that may be especially evident among IM students, given the connection between upper secondary education, employment prospects, and social inclusion in Sweden (16, 51). This context may help explain why several participants reflected on difficulties related to low self-esteem and future prospects, even though their school situation was not directly addressed in the interviews.

From the perspective of Meleis et al.'s Transition Theory (23), these reflections reveal how beliefs about self-esteem, doubts about change, and uncertainty about identity can act as constraining personal conditions during developmental transitions. For some, the perception that self-esteem is fixed—particularly among those already struggling—may limit their sense of agency and capacity for growth. At the same time, students pointed to external influences, especially social media, as shaping self-esteem through comparison and the pursuit of validation. These can be understood as constraining community and societal conditions, reinforcing insecurity and a sense of inadequacy. Furthermore, the students' background and their situation in IM may contribute to a fragile sense of identity and belonging. Despite these challenges, students also expressed hope and identified facilitating factors such as increased self-understanding, self-acceptance, and self-worth. Supporting adolescents in developing agency, clarifying their values, and reconnecting with a stronger sense of self may therefore be crucial in enabling healthier transitions—particularly for those facing educational and social vulnerabilities.

While this study aimed to describe IM students' expectations regarding *On the Inside*, their reflections point to something beyond the interactive program itself. Taken together, their narratives reveal a broader longing for self-understanding and connectedness. Fulfilling this desire—to understand one's thoughts, emotions, behaviors, and values, and to live in alignment with one's inner self—is supported by existing research as central to adolescent positive development (52, 53). The findings suggest that this inner striving may lay essential groundwork for personal growth, self-compassion, and the agency to make sustainable, wellbeing-supporting choices.

Finally, these findings must be situated within a wider societal context: the ongoing digital transition. Over the past decade, adolescence has shifted from a predominantly physical experience to an increasingly screen-based one. As such, the conditions shaping adolescents' transitions have changed—and continue to change—perhaps more rapidly than ever (54, 55). In this study, students' reflections highlight this shift as a potential constraint, limiting opportunities to “go online” with themselves and connect with their real-world surroundings. Supporting positive adolescent development, therefore, means engaging with these evolving conditions and recognizing how digital environments increasingly shape the context of growing up.

### 4.1 Strengths and limitations

A key strength of the study lies in its use of both individual interviews and focus group discussions. The individual interviews facilitated more personal and in-depth reflections, particularly on issues relating to mental health. In contrast, the focus groups supported shared meaning-making, enabling participants to expand on their thoughts in conversation with others. Although some participants were more dominant during the focus groups, this did not appear to significantly disrupt the overall conversational balance. However, a tendency toward consensus was observed, which may reflect social conformity or a reluctance to express divergent opinions (56). Nevertheless, the triangulation of data collection methods is considered to have generated a richer, more nuanced body of interview data, thereby enhancing both the credibility and transferability of the findings (25, 57).

Participant selection was guided by a purposive sampling strategy, appropriate and necessary for addressing the research aim. However, since interview participation was voluntary, there is a risk of selection bias. Students who chose to participate may have been more reflective, engaged, or interested in mental health than those who did not, potentially skewing the data toward more motivated perspectives. This may limit the transferability of the findings to less engaged students (58). At the same time, voluntary participation enabled access to students who were both willing and able to reflect on their expectations and perspectives—making their contributions especially valuable for understanding how the program resonates with its intended audience.

Another strength lies in the data analysis using RTA. This time-intensive, iterative process involved prolonged engagement and the co-construction of meaning between the researcher and participants. As a result, the findings are firmly grounded in participants' narratives, further strengthening the study's credibility (57).

## 5 Conclusion

The study reveals not only anticipated practical and psychological benefits but also provide insights into the students' lived challenges and aspirations. Rather than focusing solely on immediate expectations for the interactive program, the findings elevate the perspective to what students genuinely long for and need in order to feel better. Whether addressing physical needs, digital habits, social relationships, or questions of identity and self-esteem, they sought more than immediate solutions—expressing a desire for authentic connection with themselves. Consequently, students viewed *On the Inside* as a welcome helping hand, offering opportunities to explore their needs, rethink routines, challenge ingrained patterns, and develop a clearer sense of purpose and possibility. At its core, this shared pursuit of deeper self-understanding and connectedness was seen as laying the groundwork for more intentional choices about wellbeing and life direction.

Moreover, the students' reflections highlight the importance of supportive relationships and environments for their positive development. These findings reinforce the role of schools as valuable health-promoting arenas, offering opportunities to strengthen individual mental health and foster social cohesion in students' everyday lives—in turn supporting positive academic outcomes and future trajectories.

At the same time, it is essential to consider the broader digital transition of adolescence, which may introduce new challenges to mental health and development, as reflected in the students' narratives. Supporting positive youth development, therefore, requires active engagement with these shifting conditions and recognition of how digital environments increasingly shape the experience of growing up.

On the practical level, this study's findings could have implications for those working with students. For teachers, they highlight the value of integrating health-promoting perspectives into everyday schoolwork, such as encouraging balanced routines, mindful digital habits, and open dialogue about mental health. For school health professionals, the study underscores the importance of inviting to reflective conversations as an opportunity to support students' self-awareness, agency, and connectedness. Taken together, these insights suggest that initiatives like *On the Inside* may be most effective when embedded within a whole-school approach.

Although these findings are based on IM students, they may also be relevant to other youth populations, providing a foundation for future research to include adolescents from diverse backgrounds. Further studies should also investigate the long-term impacts of such interactive program on mental wellbeing and educational outcomes, particularly among vulnerable groups. Ultimately, this study's insights may guide the continued development of initiatives that promote adolescent mental health—investments that foster both present wellbeing and long-term development, enabling young people to participate in and contribute meaningfully to society.

## Data Availability

The raw data supporting the conclusions of this article will be made available by the authors, without undue reservation.
